# 

**Published:** 1859-11

**Authors:** 


### ORIGINAL COMMUNICATIONS. PAGE.

ART. I.—A Case of Compound Pregnancy. Reported by Dr. M. Stahl, of Hartford City, Ind. 645

ART. II.—Belladonna in Preventing the Secretion of Milk, and in Mammary Abscess. (Read before the Aurora City Medical Association, at Aurora, Ill., Oct. 3, 1859.) By A. Hard, M.D. ..... 647

ART. III.—Report of Cases in Ophthalmic Practice. By E. L. Holmes, M.D., of Chicago..... 648

ART. IV.—Report of Five Cases of Empyema. By H. Noble, M.D., of Heyworth, Ill..... 652

ART. V.—Case of Glandular Tumor of Neck. Successful Operation. By James Cody, M.D., of Watertown, Wis..... 658

ART. VI.—Diphtheria. By W. Godfrey Dyas, M.D., Chicago. (Continued.)..... 661

### BOOK AND PAMPHLET NOTICES.

Physicians' Hand Book of Practice. 1860. From S. C. Griggs & Co., Lake Street..... 673

An Introduction to Practical Pharmacy. Designed as a Text Book for the Student, and as a Guide for the Physician and Pharmaceutist; with many Formulas and Prescriptions. By Edward Parrish, Principal of the School of Pharmacy, Philadelphia, etc. Philadelphia: Blanchard & Lea. P. 720. From W. B. Keen, Lake Street..... 674

The Action of Medicines in the System; or, "On the Mode in which Therapeutic Agents introduced into the Stomach produce their peculiar effects on the Animal Economy." By Frederick William Headland, M.D., B.A., F.L.S., Licentiate of the Royal College of Physicians, etc., etc. Third edition, revised and enlarged..... 674

Address, Introductory to the Seventeenth Annual Course of Lectures in Rush Medical College, delivered Nov. 1, 1859. By J. Adams Allen, Prof. of Principles and Practice of Medicine..... 685

### EDITORIAL.

Legal Medicine in England and in Chicago..... 700

Surgical Notes.

1. Vesica-Vaginal Fistula..... 706
2. Resection of the Knee Joint..... 707
3. Congenital Absence of the Vagina, with imperfect development of the Uterus..... 707

Prospectus of the Chicago Medical Journal for 1860..... 708

Surgical Clinic of Rush Medical College..... 708

